# Crystal Structure of OXA-58 with the Substrate-Binding Cleft in a Closed State: Insights into the Mobility and Stability of the OXA-58 Structure

**DOI:** 10.1371/journal.pone.0145869

**Published:** 2015-12-23

**Authors:** Hiromichi Saino, Tomohiro Sugiyabu, Go Ueno, Masaki Yamamoto, Yoshikazu Ishii, Masashi Miyano

**Affiliations:** 1 Department of Chemistry and Biological Science, College of Science and Engineering, Aoyama Gakuin University, Sagamihara-shi, Kanagawa, Japan; 2 Advanced Photon Technology Division, RIKEN SPring-8 Center, Sayo-gun, Hyogo, Japan; 3 Department of Microbiology and Infectious Diseases, Faculty of Medicine, Toho University, Ota-ku, Tokyo, Japan; University of Cambridge, UNITED KINGDOM

## Abstract

OXA-58 is a class D β-lactamase from the multi-drug resistant *Acinetobacter baumannii*. We determined the crystal structure of OXA-58 in a novel crystal, and revealed the structure of the substrate-binding cleft in a closed state, distinct from a previously reported OXA-58 crystal structure with the binding cleft in an open state. In the closed state, the movement of three loops (α3–α4, β6–β7, and β8–α10) forms an arch-like architecture over the binding cleft through interaction between the Phe113 residues of α3–α4 and Met225 of β6–β7. This structure suggests the involvement of these flexible loops in OXA-58 substrate binding. In contrast to the mobile loops, the Ω-loop appeared static, including the conserved loop residues and their hydrogen bonds; the pivotal residue Trp169 within the Ω-loop, ζ-carbamic acid of the modified base catalyst residue Lys86, and nucleophilic residue Ser83. The stability of OXA-58 was enhanced concomitant with an increase in the hydrolytic activity catalyzed by NaHCO_3_-dependent ζ-carbamic acid formation, with an EC_50_ of 0.34 mM. The W169A mutant enzyme was significantly thermally unstable even in the presence of 100 mM NaHCO_3_, whereas the S83A mutant was stabilized with NaHCO_3_-dependent activation. The ζ-carbamic acid was shown to increase not only OXA-58 hydrolytic activity but also OXA-58 stability through the formation of a hydrogen bond network connected to the Ω-loop with Ser83 and Trp169. Thus, the static Ω-loop is important for OXA-58 stability, whereas the mobile loops of the substrate-binding cleft form the basis for accommodation of the various substituents of β-lactam backbone.

## Introduction

OXA-58 is a carbapenem hydrolyzing class D β-lactamase (CHDL) expressed by the multi-drug resistant *Acinetobacter baumannii* [1,2]. The enzyme hydrolyzes a variety of β-lactam antibiotics [1,2], including penicillins, cephalosporins, and the last resort carbapenems. The major CHDLs, such as OXA-23, OXA-24/40, OXA-51, OXA-58, and OXA-143 [3–5], have been identified from various global species of infectious bacteria [6,7]. Infectious diseases caused by the multi-drug resistant *A*. *baumannii* are difficult to treat with the currently available β-lactam antibiotics. CHDLs are thus important targets to develop an inhibitor as a novel antibiotic drug intended for combinatorial use with available β-lactams [8,9].

Structurally, β-lactamase has a loop-rich region that includes the highly conserved Ω-loop. These loop structures have multiple functions, such as determining substrate specificity and β-lactamase stability [10–15]. Studies of class D β-lactamase crystal structures have largely focused on the architecture of the substrate-binding clefts formed by the loops. The substrate-binding clefts have a variety of widths and shapes, and the variable features of these clefts have been proposed to be relevant to the extended substrate specificities of the β-lactamases [16–20]. However, the relationships between cleft features and substrate specificity have not been fully explored based on the structural differences in CHDLs. A crystallographic study of OXA-58 as a carbapenemase was recently reported, and the binding cleft was wider than that reported for other CHDLs because of an open conformation of loops [21]. In contrast, the OXA-24 carbapenemase has a narrow, short, tunnel-like cleft composed of closed loops [19]. Differences in open and closed conformations could result from distinct amino acid components or movement of the loops. Crystal structures of OXA-58 with different crystal packings could allow the verification of loop mobility.

The Ω-loop of the class D β-lactamases contains a pivotal Trp residue that contributes to ζ-carbamic acid formation on the Lys base catalyst residue. The Lys nitrogen atom (N^ζ^) is spontaneously modified by *N* ^ζ^-carboxylation in the presence of the carboxy donor NaHCO_3_. The resultant ζ-carbamic acid has been confirmed by ^13^C-NMR [1,22] and crystal structures of class D β-lactamases [17,20,21,23]. OXA-58 hydrolytic activity is increased by up to 7-fold when ζ-carbamic acid is present, increasing its catalytic efficiency for penicillins (ampicillin, oxacillin, amoxicillin, benzyl penicillin, and carbenicillin), the cephalosporin cephalothin, and the carbapenem imipenem [1]. The activation of class D β-lactamases increases bacterial drug resistance. Indeed, NaHCO_3_ supplied in medium increases the minimum inhibitory concentration of *E*. *coli* transformed with OXA-1 [24]. The formation of ζ-carbamic acid depends on both the pH and concentration of NaHCO_3_ [22,25]. The arterial blood typically containing 24 mM HCO_3_^−^ ion [26], which is sufficient to activate class D β-lactamases [1,22]. The ζ-carbamic acid in class D β-lactamases is an important feature for understanding drug resistance and the high mortality from bloodstream infections caused by *A*. *baumannii* [27].

The increase of hydrolytic activity with ζ-carbamic acid formation has been well studied in class D β-lactamases, whereas its effect on protein stability has received little attention. The ζ-carbamic acid may also be involved in the stability of CHDLs because it forms hydrogen bonds in the active site with the Trp residue of the conserved Ω-loop [18,21,23]. In the present study, we have determined a crystal structure of OXA-58 with novel protein packing and evaluated the organization of the flexible loop regions in comparison with previously reported OXA-58 crystal structures (PDB ID: 4OH0) [21]. Our structure showed that the Ω-loop has a static structure that is important for the stability of the OXA-58 structure. We measured the thermal stability of OXA-58 to elucidate the contribution of the ζ-carbamic acid to protein stability with mutations introduced in the residues involved in ζ-carbamic acid formation.

## Materials and Methods

### OXA-58 expression and purification

β-Lactamase OXA-58 was cloned from *A*. *baumannii* [28] and N-terminal truncated OXA-58Δ33 and Δ43 were inserted into the *pCold* ^TM^ *I* expression vector (TAKARA), which contains a His-tag and a translation-enhancing sequence. The Δ43 construct was generated using the synthetic primer 5′-CATATCGAAGGTAGGGTTCAAGCGCTTTTTAATGA-3′ for N-terminal truncation. Plasmid vectors were transformed into BL21 Star^TM^ (DE3) pLysS competent cells (Life Technologies). OXA-58s were expressed using the transformed BL21 cells in LB medium supplemented with 100 μg/ml (w/v) ampicillin, with expression induced by the addition of 0.5 mM IPTG for 18 h at 15°C. Cells were collected, sonicated in 50 mM MES (pH 6.5), 1 mM EDTA, 20 mM NaHCO_3_ on ice, and the pellets were removed by centrifugation (12000 rpm, 20 min) at 4°C. The supernatant was suspended in Ni-Sepharose Fast 6 (GE Healthcare) by gently mixing the solution on a rotator for 1 h at 4°C in a column. The resin was successively washed with a buffer containing 50 mM HEPES (pH 7.5), 500 mM NaCl, 50 mM imidazole, and 20 mM NaHCO_3_, and then OXA-58 was eluted from the column with 500 mM imidazole. The eluate was desalted in 50 mM HEPES (pH 7.5) with 20 mM NaHCO_3_ and PD-10 (GE Healthcare).

### Crystallization and data collection

The N-terminal truncation of 43 residues (OXA-58Δ43) created a higher resolution crystal than OXA-58Δ33. OXA-58Δ43 was prepared in the same manner as described above, with subsequent size exclusion chromatography carried out on a Superose 12 column (GE Healthcare) at 4°C with additional purification and buffer exchange to 50 mM MOPS (pH 7.0) with 100 mM NaHCO_3_. The purified protein was concentrated to 10 mg/ml using centrifugal concentration (Vivaspin 10k, GE Healthcare) and was crystallized using a sitting-drop vapor diffusion method by mixing each 1 μl of protein solution and reservoir solution (0.1 M MOPS pH 7.0, 1.5 M Li_2_SO_4_). Thin plate crystals grew in 2 weeks at 24°C only when in the presence of NaHCO_3_ in the protein solution. On the other hand, OXA-58Δ33 formed a large hexagonal crystal, but the shape was distorted and less diffractive. Crystal screening was carried out using a MicroMax rotating anode diffractometer equipped with RAXIS VII (Rigaku). These established conditions were repeated for all data collection.

Crystals were transferred into a cryoprotectant solution (15% glycerol, 0.1 M MOPS pH 7.0, 1.6 M Li_2_SO_4_) and dipped in liquid nitrogen on a cryoloop. The cryo-cooled crystals were sent to SPring-8, and data collection was carried out using a remote operation system at BL26B1 [29], which allows to control all instruments (e.g., the sample changer, goniometer, detector, beam shutter) *via* the Internet. All diffraction data was collected by 5.0 sec exposure at 1.00000 Å with 360 images collected at 1° oscillation.

### Structure determination and refinement

Diffraction images were processed using MOSFLM [30] and scaled and merged up to 1.8 Å resolution with SCALA/CCP4 [31] as space group *R3*. Molecular replacement using Phaser [32] as a twin crystal (0.46 twin fraction) in Phenix [33] produced a monomer in an asymmetric unit (search model PDB ID: 4HO0). Subsequent refinement was carried out using phenix.refine [34–36]. The phasing after rigid body refinement gave clear electron densities, but fragmented electron densities in the loop-rich regions suggested different loop structures distant from the search model. Simulated annealing improved the loop structures and refinement statistics with well-fitted electron densities. After manual model rebuilding on COOT [37] and iterated cycles of restrained refinement with water picking, the final *R* and *R*_free_ values were reduced to 0.148 and 0.162, respectively. The statistics for data collection and structure refinements are shown in Table 1. The figures were prepared with PyMOL (Schrödinger, LLC) [38,39].

**Table 1:** Date collection and refinement statistics for the OXA-58 crystal structure.

**Date collection**	
Space group	R3
Cell dimensions	
*a*, *b*, *c* (Å)	75.3, 75.3, 120.7
*α*, *β*, *γ* (°)	90, 90, 120
Resolution range (Å)	28.7–1.8 (1.9–1.8)*
*R*_merge_ (%)	7.3 (27.1) *
*I/*σ*I*	6.9 (2.8) *
Completeness (%)	99.6 (99.5) *
Multiplicity	2.7 (2.7) *
**Refinement**	
Resolution (Å)	1.8
*R*/*R*_free_	0.149/0.169
No. of atoms	
Protein	1896
Water	148
B-factors	
Protein	19.4
Water	24.0
RMSD	
Bond length (Å)	0.007
Bond angles (°)	1.120
**PDB id**	5BOH

*The highest shell statistics are reported in parentheses.

### Site-directed mutagenesis

Amino acid substitutions were introduced into the OXA-58Δ33 construct in *pCold* ^TM^ *I* using Q5 High-Fidelity DNA Polymerase (NBE) and the synthetic primers: W169A, 5′-GTTGATCAATTTGCGTTGAAAGGGCCT-3′; W169F, 5′-GTTGATCAATTTTTCTTGAAAGGGCCT-3′ and S83A, 5′- GCTTATATTCCTGCAGCGACATTTAAAATTGCC-3′. The polymerase chain reaction was performed for the entire ~5 kb plasmid, and the mutated plasmids were isolated by Dpn I digestion and amplified by transforming DH5α cells followed by plasmid extraction. The mutant plasmids were transformed into BL21 cells after sequence verification, and the mutant enzymes were prepared as described above.

### Effect of NaHCO_3_ concentration on OXA-58 activity and stability

OXA-58Δ33 (wild-type) and its W169A and S83A mutant activities were measured colorimetrically using nitrocefin as the substrate. The purified enzymes were desalted by PD-10 (GE Healthcare) to remove NaHCO_3_, and the stock enzyme concentration was prepared to 10 μg/ml in 50 mM MOPS (pH 7.0). All solutions were prepared using degassed water containing 1 mM glutamic acid (pH 3) to remove previously dissolved carbonate and carbon dioxide, and the NaHCO_3_ concentrations were determined by adding 1 M NaHCO_3_ solution. Final concentrations in 200 μl reaction mixtures were 0.2 M sodium phosphate (pH 7.0), 125 mM–0.7 μM NaHCO_3_, 25 μM nitrocefin, and enzyme (protein concentration: 0.025 μg/ml for wild type, 0.125 μg/ml for W169A, and 5 μg/ml for S83A). The hydrolyzed nitrocefin product was quantified as absorbance at 485 nm (ε = 20,500 M^−1^cm^−1^) on a Fusion α microplate reader (Packard). The reaction was initiated by adding the premixed buffer solution to the enzyme solutions, and the absorbance was recorded for 25 min at room temperature. The activities were given as the initial velocities of linear regression of the progress curve. The EC_50_ values were estimated by nonlinear regression curve fitting to a logistic equation using Gnuplot 5.0 [40]. The decomposition rates of nitrocefin in non-enzyme controls were less than 3.5 × 10^−9^ (μmol/sec) at any NaHCO_3_ concentrations.

Thermal stabilities of the enzymes were evaluated as the residual activity after heat treatment. The desalted enzymes were adjusted to 100 μg/ml in 0.2 M MOPS (pH 7.0) with 0.01 mM, 1 mM, and 100 mM NaHCO_3_; heated for 1 h at 50°C; and then immediately cooled down to 4°C under temperature control on a thermal cycler. The heat-treated enzymes were diluted with 0.2 M MOPS (pH 7.0) containing 100 mM NaHCO_3_ on ice, and the enzyme activities were measured in the presence of 100 mM NaHCO_3_ as described above.

## Results

### The closed substrate-binding cleft

**Fig 1:**
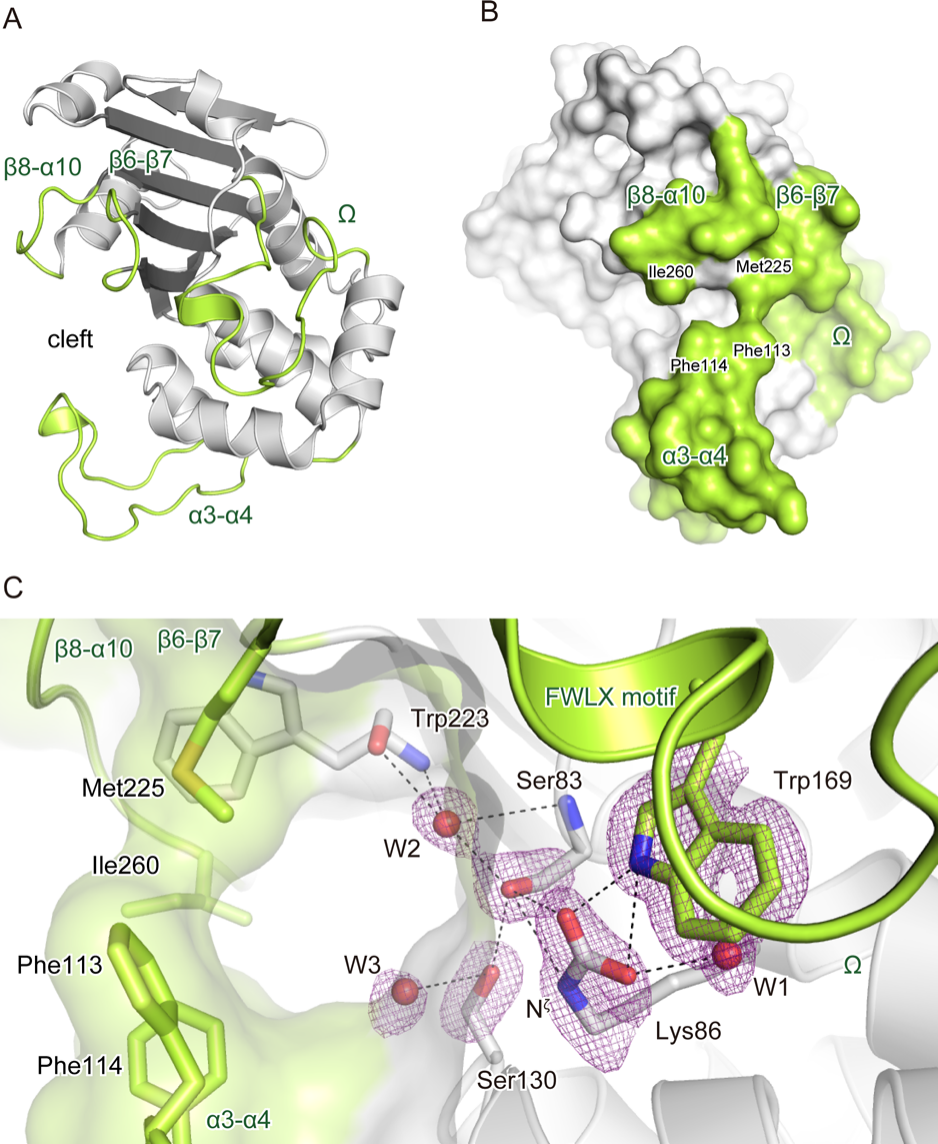
The closed substrate-binding cleft of OXA-58. A, A representation of the overall OXA-58 structure. The loops forming the substrate-binding cleft are colored green. B, A representation of the overall OXA-58 surface shown in the same colors as in panel A. C, The hydrogen bond network in the active site. Dashed line connections in stick models indicate hydrogen bonds between each atom from the ζ carbamic acid of Lys86 and Ser83. The purple meshes are a *F*_o_−*F*_c_ omit map of the residues involved in the hydrogen bond network that includes bound water molecules with contouring at the 3*σ* level. The arch-like roof architecture is represented using a transparent molecular surface envelope, and the residues forming the architecture are shown in the stick model.

The crystal structure of OXA-58 showed the typical folding of a class D β-lactamase and consisted of six long β-strands and eight α-helices (Fig 1A). The substrate-binding cleft between the two domains was a cavity in the loop-rich region that contained four loops: α3–α4 (97–122), Ω-loop (155–177), β6–β7 (225–230), and β8–α10 (254–260). Except for Ω-loop, the loops showed significantly different orientations from the previously reported structure [21] and formed a narrower substrate-binding cleft. The β6–β7 and β8–α10 loops lay along one side of the substrate-binding cleft to form a wall, with the α3–α4 loop forming the opposite wall (Fig 1B). The cleft had an arch-like architecture formed by Phe113 and Phe114 of α3–α4, Met225 of β6–β7, and Ile260 of β8–α10 (Fig 1B and 1C), with the side chains of Phe113 and Met255 forming a roof over the cleft at a 4.2 Å distance. The Ω-loop was attached to a side end of the cleft through a short α-helix motif (Phe^168^–Trp^169^–Leu^170^–Lys^171^), and the indole ring of Trp169 in the Ω-loop was inserted into the active site.

The active site architecture was well defined, with clear electron densities of the individual amino acid residues and bound water molecules (Fig 1C), which showed that the residues formed a network of hydrogen bonds. The side chain Ν^ζ^ of Lys86 was carboxylated by NaHCO_3_ supplied during purification and crystallization. An oxygen atom of ζ-carbamic acid formed a bifurcated hydrogen bond with the nucleophile hydroxyl oxygen of Ser83 and the indole-NH nitrogen of Trp169 in the Ω-loop at distances of 2.6 Å and 2.9 Å, respectively. The other oxygen atom of the *N* ^ζ^-carboxy group was within 3.2 Å of Trp169-NH and 2.6 Å of the bound water (W1). The carbamic–NH acid nitrogen of Lys86 was located within a hydrogen bond distance of 3.2 Å from the oxygen of Ser83-OH. Thus, the hydrogen bond of the ζ-carbamic acid group and Trp169 formed a link between the active site and the Ω-loop. This network of hydrogen bonds was extended *via* the Ser83-OH to bound waters and a conserved Ser130 (Fig 1C). The other water molecule (W2) was at 2.5 Å to the oxygen of Ser83-OH, and additional three hydrogen bonds were with two of the -NH and a -CO groups on the Ser83 and Trp223 backbones, respectively, at the W2 position, which was presumed to be the accepting site for the emerging oxyanion formed in the hydrolytic reaction [18,41]. The other conserved Ser130 formed a hydrogen bond with Ser83-OH and another water molecule (W3) at distances of 3.2 Å and 3.4 Å, respectively, from each of the hydroxyl oxygen of Ser83.

### Stabilization of OXA-58 by addition of NaHCO_3_

**Fig 2:**
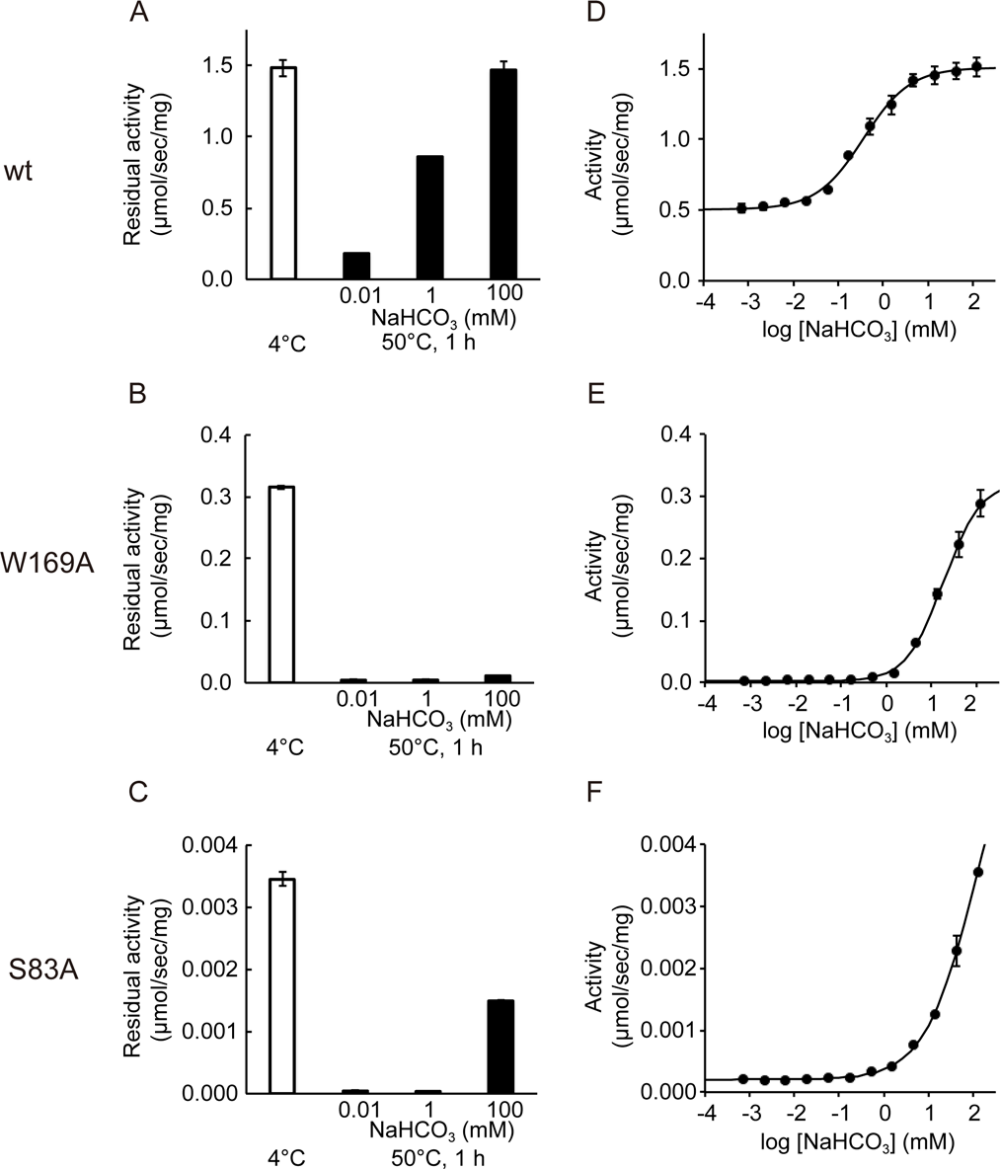
Stabilization of OXA-58 concomitant with activation in NaHCO_3_. The bar graphs in the left column show the residual activities of wild-type (wt, A), W169A (B), and S83A (C) after heat treatment. The white bars represent the specific activities of enzymes stored at 4°C without heat treatment in 100 mM NaHCO_3_ as a control. The black bars are the residual activities after heat treatment in presence of NaHCO_3_ at the concentrations indicated. The graphs of plots and curves in the left column are the specific activities of the wild-type (D), W169A (E), and S83A (F) enzymes vs. NaHCO_3_ concentrations on a logarithmic scale.

To estimate the contributions of the hydrogen bond to OXA-58 protein stability, the residual activities of the wild-type, W169A, and S83A proteins were assayed after 1 h of heat treatment at 50°C in the presence of varied concentrations of NaHCO_3_ (Fig 2A–2C). The NaHCO_3_ concentrations were established based on the activation curve of the wild-type enzyme (Fig 2D) at the lowest (0.01 mM), partially activated (1 mM), and saturated (100 mM) concentrations. Wild-type OXA-58 sustained 99% of full activity after heat treatment with 100 mM NaHCO_3_ and indicated an enhancing effect of ζ-carbamic acid on OXA-58 protein stability (Fig 2Α). The residual activity was reduced to 58% and 12% with 1 and 0.01 mM NaHCO_3_, respectively, suggesting that the OXA-58 protein becomes unstable in the absence of the ζ-carbamic acid. In contrast, W169A was significantly unstable with heat treatment, even at 100 mM NaHCO_3_ (Fig 2Β) The residual activity of W169A was 1% in 0.01 mM and 1 mM of NaHCO_3_ and 4% in 100 mM of NaHCO_3_. Remarkably, at 100 mM NaHCO_3_, W169A showed the increased activity caused by the ζ-carbamic acid formation (Fig 2Ε), but almost no stabilizing effected was observed (Fig 2Β). These results suggest that the hydrogen bond residue Trp169 is crucial for the thermal stability of OXA-58. On the other hand, S83A was reasonably stabilized with 100 mM NaHCO_3_ in comparison with the wild-type OXA-58, with 43% residual activity retained (Fig 2C) concomitant with the activation curve (Fig 2F). The significant instability of W169A indicated that the hydrogen bond formation between ζ-carbamic acid and Trp169 substantially contributes to protein stability through hydrogen bond network formation. W169F was also unstable even in the presence of NaHCO_3_ (S1 Fig), indicating that the loss of hydrophobic interaction of Trp169 does not contribute to the destabilization of the W169A mutant.

Wild-type OXA-58 was activated 3-fold in a saturable manner with an increase in NaHCO_3_ concentration from 0.7 μM to 125 mM (Fig 2D), which is consistent with ζ-carbamic acid formation at Lys86 in the crystal structure with NaHCO_3_ as an additive (Fig 1C). The activities of S83A and W169A were retained at 1/500 and 1/5, respectively, of the wild-type enzyme at 125 mM NaHCO_3_, and both the mutants were also activated in accordance with an increase in NaHCO_3_, indicating that ζ-carbamic acid formed on Lys86 in both the mutants as well (Fig 2E and 2F). It is noteworthy that the hydrolytic activities of both mutants in the absence of NaHCO_3_ were very low, whereas the addition of NaHCO_3_ resulted in ten to hundred fold increase from 0.00020 to 0.0035 μmol/sec/mg protein and 0.0028 to 0.29 μmol/sec/mg protein for S83A and W169A, respectively. The activation profiles were typical sigmoidal curves in the wild-type and mutant enzymes. The calculated EC_50_ was 0.36 mM with a Hill coefficient of 0.84 in the non-linear fitting (Fig 2A). The curves for the mutant enzymes were shifted to a more than 10-fold higher EC_50_ concentration over 10 mM, EC_50_ = 113 mM, and 19 mM for S83A and W169A, with Hill coefficients of 0.76 and 1.1, respectively (Fig 2E and 2F). These results suggest that Ser83 and Trp169 in the hydrogen bond network facilitate ζ-carbamic acid formation on Lys86 in the activated form of Lys86.

## Discussion

### Flexible loops for substrate binding

**Fig 3:**
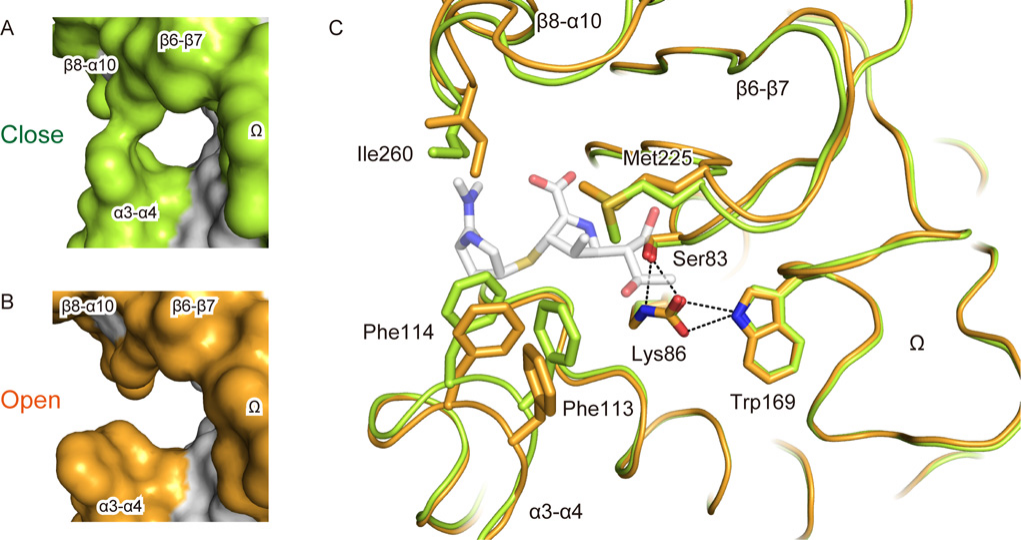
Crystal structures of apo OXA-58 with a static Ω-loop and the flexible loops of the substrate-binding cleft. Left, the surface representation of the substrate-binding cleft in closed state (A, green), and open state (B, orange) in a previously reported OXA-58 crystal structure (PDB ID: 4OH0). Right, superimposed models of the two crystal structures of OXA-58; closed state (green) and open state (orange). The dashed lines represent hydrogen bonds for the ζ-carbamic acid of Lys86 connect to the Ω-loop from the loop containing Ser83. The white model represents a acyl-intermediate meropenem structure superimposed with OXA-23 (PDB ID: 4JF4) as a prospected carbapenem binding.

The crystal structures of OXA-58 were compared between protein packing in trigonal (the present study) and orthorhombic [21] crystals (Fig 3A–3C). The loops α3–α4, β6–β7, and β8–α10 are flexible and mobile around the substrate-binding cleft as indicated by the up to 4-fold higher root-mean-square deviation (RMSD) compared with the overall average 0.49 Å RMSD. The flexible loops resulted in the significant displacement of the aromatic ring positions in Phe113 and Phe114 by 2–3 Å. Ile260 and Met225 showed conformational changes accompanied with movement of the β8–α10 and β6–7 loops, in which the end methyl groups of Met225 and Ile260 were rotated toward Phe113 and Phe114, respectively, to form the arch-like roof structure over the cleft (Fig 1B and 1C). The water structure in the narrow closed cleft was different from that in open state [21]. Except for the conserved water W1 and W2 (Fig 1C), the water molecules including W3 and its vicinity were arranged in more than 1 Å distant positions from those in the open state.

The substrate-binding cleft of OXA-58 has a variable width, which could be responsible for the extended spectrum of OXA-58 enzymatic activity. The crystal structure of carbapenemase OXA-24 [19] has superimposable catalytic architectures of a closed substrate-binding cleft (S2 Fig), with the arch of the tunnel formed by Tyr112 and Met223, which is similar to the interaction between Phe113 and Met225 in our OXA-58 crystal structure. Superimposing the OXA-58 model with an acyl-intermediate carbapenem of OXA-23 [41] indicates that the *S*-linked substituent of pyrrolidine is located between the moving amino acids Phe114 and Ile260 (Fig 3) and that the 6-hydroxyethyl substituent of the β-lactam ring is located between Phe113 and Met225. These substituents are highly variable parts in the chemical structures of a variety of β-lactam antibiotics. Therefore, the movements of the amino acid residues Phe113, Phe114, Met225, and Ile260 (S1 Video), which are induced-fit components of the substrate-binding cleft, could facilitate the accommodation of diverse substituents of β-lactam antibiotics, including carbapenems. Indeed, the oxacillin complex structure of OXA-24 shows induced fitting of the β6–β7 loop conformation through binding and makes the cleft wider to accept the large side chain of oxacillin [42].

### Static Ω-loop connected to the hydrogen bond network

Both crystal structures show the ζ-carbamic acid of the base catalyst Lys86 and the hydrogen bond network between ζ-carbamic acid, Ser83, and Trp169 as almost perfectly superimposable within the active site (Fig 3), with the long loop element Ω-loops also superimposable with no conformational change. The averaged RMSD was 0.25 Å in the Ω-loop, which was significantly lower than the overall RMSD by 0.49 Å, thus indicating that the Ω-loop structure is static. The hydrogen bond network of the ζ-carbamic acid on Lys86 is essential for enhancing the stability of the OXA-58 protein as well as an increase in its hydrolytic activity. The ζ-carbamic acid hydrogen bond network sufficiently lowers the EC_50_ and increases the activity under physiological conditions [26]. Both hydrolytic activity and protein stability as well as architecture of substrate-binding cleft could be crucial for the acquisition of OXA-58 multidrug resistance independent of host factors [2,5]. The tandem catalytic residues Ser and *N* ^ζ^-carboxylated Lys accompanied by the hydrogen bond network with Trp on the Ω-loop are the common architectural elements in class D β-lactamases.

The less than unity Hill coefficients for wild-type and S83A OXA-58 could reflect the multi-step process of ζ-carbamic acid formation, whereas that of W169A was almost unity (S3 Fig). Before *N* ^ζ^-carboxylation, the carboxy position in the uncarboxylated Lys86 could be occupied by a surrounding water molecule. In the crystal structure of OXA-10, there was a bound water molecule between the corresponding Trp and uncarboxylated Lys (PDB ID: 1FOF) [20]. Given the water in the decarboxylated site, ζ-carbamic acid formation on Trp169 could require the following two steps (S4 Fig): first, dehydration of the bound water between Lys86 and Trp169, followed by the *N* ^ζ^-carboxylation of Lys86 with carbon dioxide. Noticeably, the base catalyst Lys86 without ζ-carbamic acid possesses sufficient activity (Fig 2D), suggesting that the bound water could represent the deacylation nucleophile activated by the sandwiched hydrogen bond of the side-chain amine on Lys86 and the aromatic indole-NH of Trp169 in the hydrophobic milieu of the OXA-58 active site.

## Supporting Information

S1 Fig Stability of W169F mutant. (TIF)

S2 Fig Superimposed structure of OXA-58 and OXA-24 in closed state. (TIF)

S3 Fig Hill plot of W169A exhibits distinct mode of ζ-carbamic acid formation from wild-type and S83A. *θ* is the fraction of activation (0–1) under each NaHCO_3_ concentrations, where the minimum (*θ* = 0) and maximum (*θ* = 1) activities were estimated from the curve fittings represented in Fig 2A–2C.
(TIF)

S4 Fig Scheme 1. (TIF)

S1 Video The movement of flexible substrate-binding cleft of OXA-58 represented with interpolated trajectory between two crystal structures. The trajectory was generated by PyMOL version 1.7 [38,39] with opened structure (4HO0) [21] and closed structure (5BOH, in this work). The color representations are identical to that in Fig 1, and added meshes to display sphere models of side chain atoms with Van der Waals radii.
(MOV)
