# Supplementary figures and images for: Crystal Structure of OXA-58 with the Substrate-Binding Cleft in a Closed State: Insights into the Mobility and Stability of the OXA-58 Structure
Source: PLoS One. 2015 Dec 23;10(12):e0145869. doi: 10.1371/journal.pone.0145869 (PMC4689445; doi:10.1371/journal.pone.0145869)

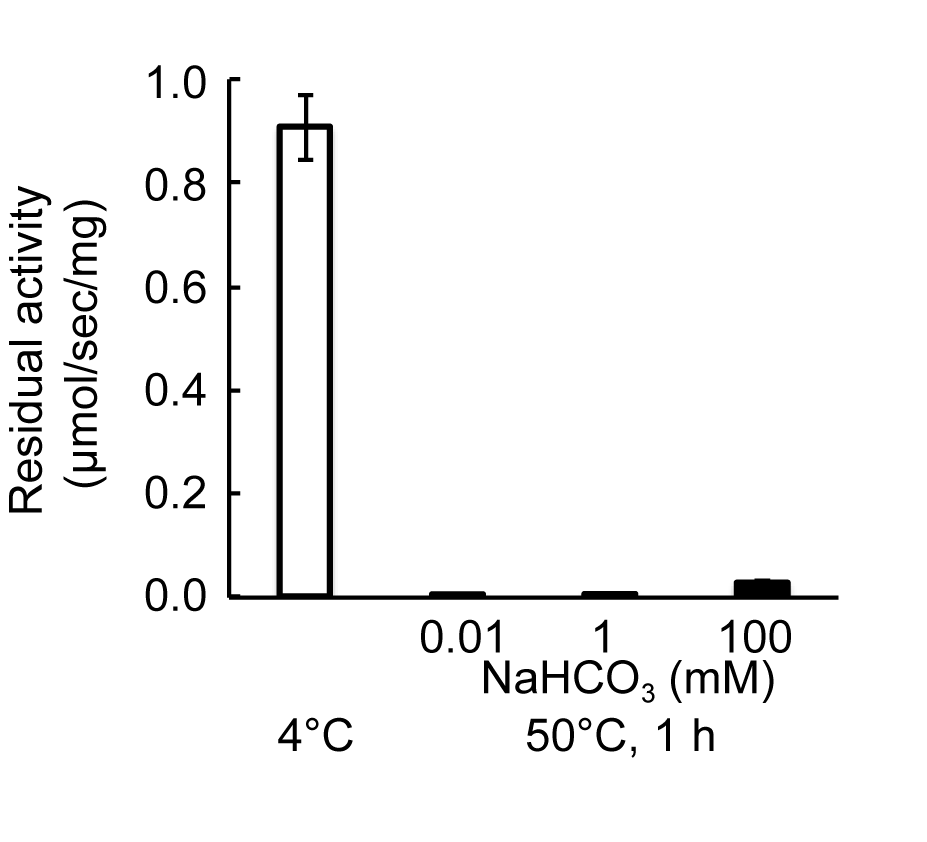

Supplement: S1 Fig — (TIF) [file pone.0145869.s001.tif]

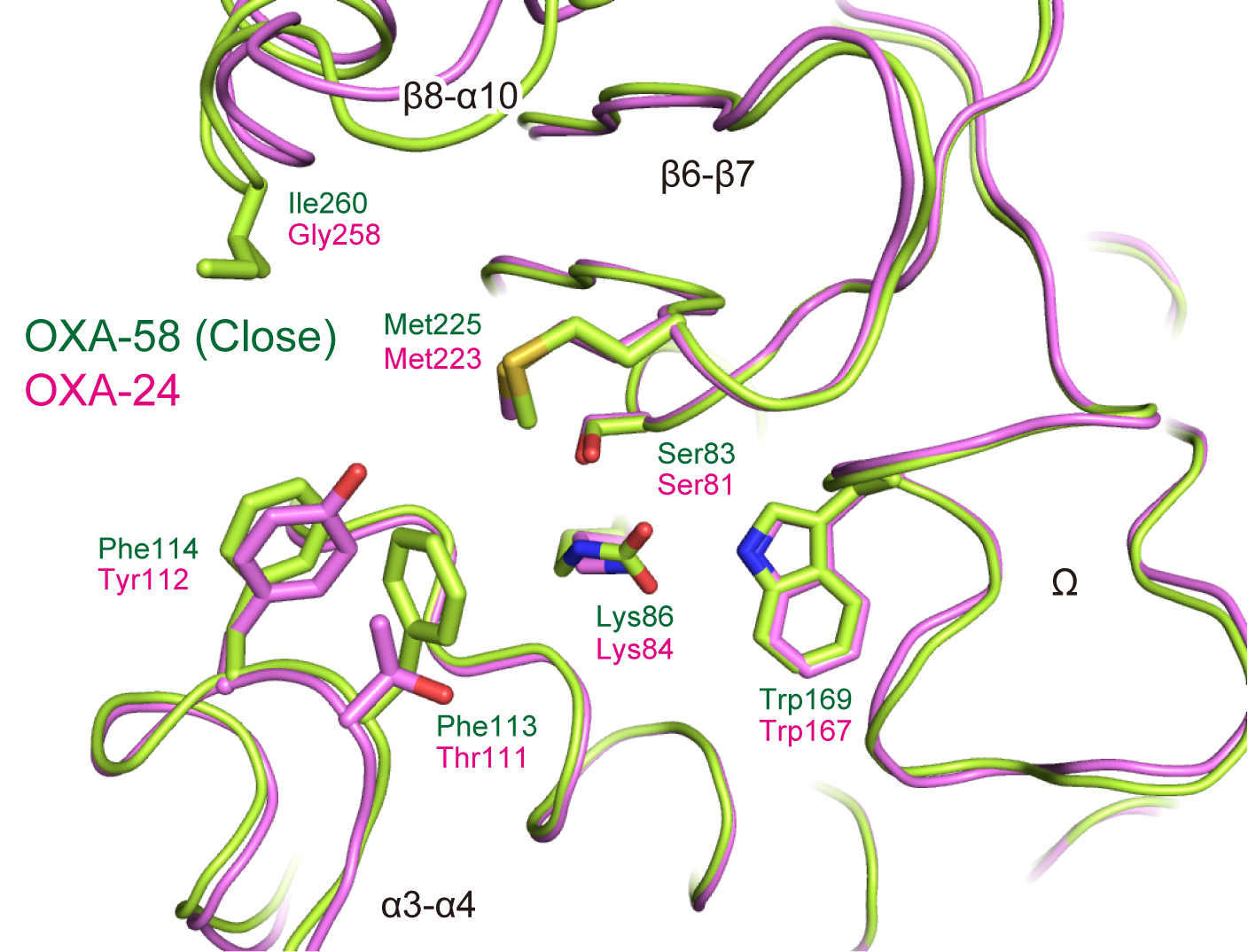

Supplement: S2 Fig — (TIF) [file pone.0145869.s002.tif]

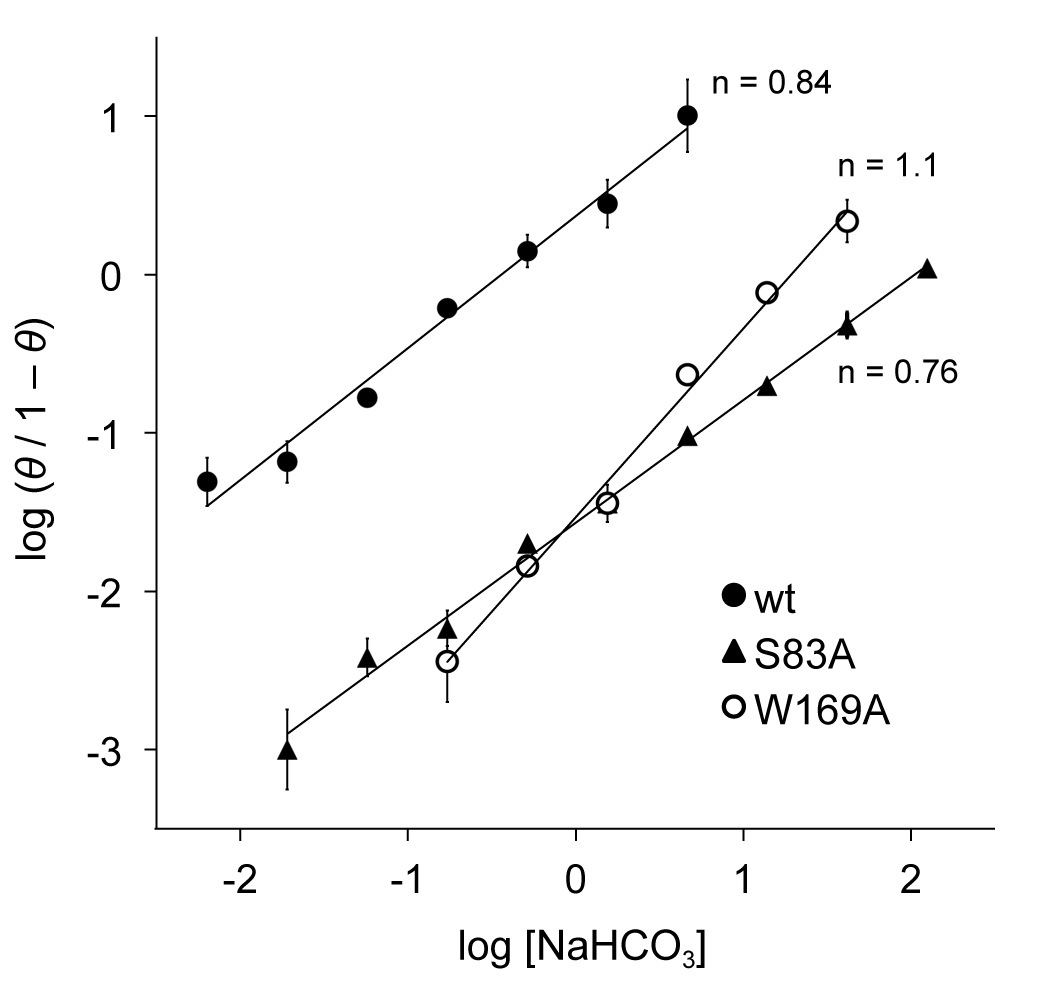

Supplement: S3 Fig — θ is the fraction of activation (0–1) under each NaHCO3 concentrations, where the minimum (θ = 0) and maximum (θ = 1) activities were estimated from the curve fittings represented in Fig 2A–2C. (TIF) [file pone.0145869.s003.tif]

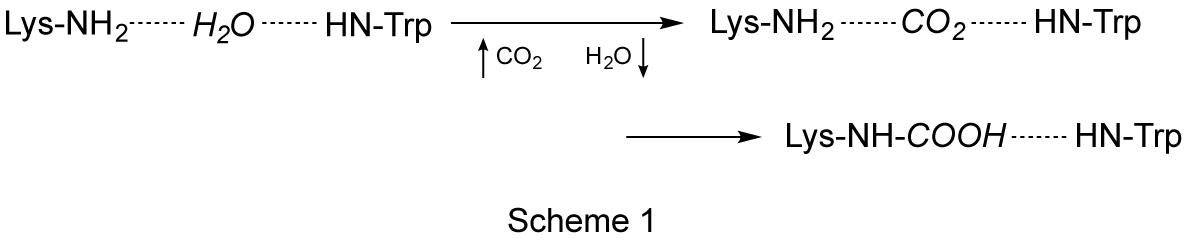

Supplement: S4 Fig — (TIF) [file pone.0145869.s004.tif]
